# Body mass index, triglyceride-glucose index, and prostate cancer death: a mediation analysis in eight European cohorts

**DOI:** 10.1038/s41416-023-02526-1

**Published:** 2023-12-12

**Authors:** Josef Fritz, Sylvia H. J. Jochems, Tone Bjørge, Angela M. Wood, Christel Häggström, Hanno Ulmer, Gabriele Nagel, Emanuel Zitt, Anders Engeland, Sophia Harlid, Isabel Drake, Pär Stattin, Tanja Stocks

**Affiliations:** 1 Department of Translational Medicine, Lund University, Malmö, Sweden; 2 Institute of Medical Statistics and Informatics, Medical University of Innsbruck, Innsbruck, Austria; 3 Department of Clinical Sciences Lund, Lund University, Lund, Sweden; 4 Department of Global Public Health and Primary Care, University of Bergen, Bergen, Norway; 5 Cancer Registry of Norway, Oslo, Norway; 6 Department of Public Health and Primary Care, University of Cambridge, Cambridge, UK; 7 Northern Registry Centre, Department of Public Health and Clinical Medicine, Umeå University, Umeå, Sweden; 8 Agency for Preventive and Social Medicine (aks), Bregenz, Austria; 9 Institute of Epidemiology and Medical Biometry, Ulm University, Ulm, Germany; 10 Department of Internal Medicine 3, LKH Feldkirch, Feldkirch, Austria; 11 Vorarlberg Institute for Vascular Investigation and Treatment (VIVIT), Feldkirch, Austria; 12 Department of Chronic Diseases, Norwegian Institute of Public Health, Bergen, Norway; 13 Department of Radiation Sciences, Oncology, Umeå University, Umeå, Sweden; 14 Department of Clinical Sciences Malmö, Lund University, Malmö, Sweden; 15 Department of Surgical Sciences, Uppsala University, Uppsala, Sweden

**Keywords:** Prostate cancer, Risk factors, Cancer epidemiology

## Abstract

**Background:**

Insulin resistance is a hypothesised biological mechanism linking obesity with prostate cancer (PCa) death. Data in support of this hypothesis is limited.

**Methods:**

We included 259,884 men from eight European cohorts, with 11,760 incident PCa’s and 1784 PCa deaths during follow-up. We used the triglyceride-glucose (TyG) index as indicator of insulin resistance. We analysed PCa cases with follow-up from PCa diagnosis, and the full cohort with follow-up from the baseline cancer-free state, thus incorporating both PCa incidence and death. We calculated hazard ratios (HR) and the proportion of the total effect of body mass index (BMI) on PCa death mediated through TyG index.

**Results:**

In the PCa-case-only analysis, baseline TyG index was positively associated with PCa death (HR per 1-standard deviation: 1.11, 95% confidence interval (CI); 1.01–1.22), and mediated a substantial proportion of the baseline BMI effect on PCa death (HR_total effect_ per 5-kg/m^2^ BMI: 1.24; 1.14–1.35, of which 28%; 4%–52%, mediated). In contrast, in the full cohort, the TyG index was not associated with PCa death (HR: 1.03; 0.94-1.13), hence did not substantially mediate the effect of BMI on PCa death.

**Conclusions:**

Insulin resistance could be an important pathway through which obesity accelerates PCa progression to death.

## Introduction

The association of obesity with prostate cancer (PCa) incidence and death is double-edged. Obesity is associated with lower risk of diagnosis of low-risk, commonly screen-detected PCa, but is not associated with the risk of more advanced PCa [1–7]. In contrast, obesity has consistently been associated with worse PCa prognosis and PCa-specific death [1, 3–5, 8–17], but the mechanisms underlying these associations remain unclear. One possible mechanism is that less effective PCa treatment of obese men results in a higher risk of disease recurrence [1, 18, 19], but biological mechanisms may also be in action [1, 8, 20–22]. In support of biological mechanisms is the fact that obesity is associated with an increased risk of PCa progression and death also when studying only low-risk PCa’s [3, 23]; findings which are less likely to be affected by curative and palliative treatment.

Insulin resistance and the resulting hyperinsulinemia is one potential biological pathway connecting obesity with progression and death from PCa [1]. Whilst hyperinsulinemia has not been associated with the diagnosis of PCa in prospective cohort studies [24, 25], there is evidence for its involvement in PCa progression and death [26, 27]. For example, in the Physicians’ Health Study, plasma concentrations of prediagnostic C-peptide – a robust marker of plasma insulin levels - were positively associated with higher PCa-specific mortality [26].

The product of fasting triglyceride and glucose levels (triglyceride-glucose [TyG] index) is a simple measure of insulin resistance [28]. It correlates well with the euglycemic-hyperinsulinemic clamp test, the gold standard for determining insulin resistance, and has validity similar to the frequently used homoeostatic model assessment insulin resistance index [29]. Both lipotoxicity and glucotoxicity play crucial roles in insulin resistance modulation, and both are reflected in the TyG index [30, 31].

Prospective epidemiological studies quantifying (i) the association of insulin resistance and death from PCa, and (ii) how much of the effect of obesity on higher risk of PCa death is mediated through insulin resistance are lacking. This study aimed at estimating these quantities using body mass index (BMI) and TyG index measurements in pooled data of eight European population-based cohorts.

## Materials and methods

### Study population

This study pooled data from four Swedish, three Norwegian, and one Austrian population-based cohorts. Information was obtained from health examinations, performed between 1972 and 2016, including height and weight as measured by medical staff, glucose and triglyceride values from blood draws, and questionnaire-assessed smoking status. The study was approved by research ethics committees in the respective countries. Further details on the cohorts can be found in the Supplementary Information, Supplementary Materials and Methods.

### Follow-up and endpoint assessment

To obtain information on cancer diagnoses, date and cause of death, and date of emigration (not available for the Austrian cohort), each cohort was linked to the respective national Cancer Register, Cause of Death Register, and Population Register. Study participants were followed until emigration, death, or end of study (2012 for the Norwegian, 2014 for the Austrian, and 2016 for the Swedish cohorts). PCa cases were identified in the cancer registers using International Classification of Diseases, version 7 code 177 and/or ICD-10 code C61. For the Swedish cohorts, additional linkages of the data with the Longitudinal Integration Database for Health Insurance and Labour Market Studies (LISA), the Patient Register, and the National Prostate Cancer Register (NPCR) were performed (see also Supplementary Information, Supplementary Materials and Methods).

### Exclusions

From the original dataset, observations were excluded for a variety of reasons, including men with inconsistencies in cancer and death dates and/or a cancer diagnosis occurring before the baseline visit (except non-melanoma skin cancer), visits with incomplete, inconsistent, or implausible reporting, and visits occurring before the age of 21. After exclusions, 259,884 out of originally 431,525 men were left for our final analysis population (Figure S1). The vast majority of these exclusions were due to missing information on glucose levels in the Norwegian cohorts during years when it had not been routinely measured. Additional details are provided in the Supplementary Information, Supplementary Materials and Methods.

### Statistical analysis

Exposure (BMI) and mediator (TyG index) values were taken from each man’s baseline visit. BMI was calculated as [weight (kg)/height (m)^2^], and the TyG index as ln[triglycerides (mg/dl) × blood glucose (mg/dl)/2] [28]. TyG index values were standardised (z-transformed) separately for cohort and fasting status (<8 h vs. ≥8 h) to account for effects of fasting state and cohort-specific differences, such as analytical methods. Tests of interaction between BMI and cohort, and TyG index and cohort, did not indicate differential associations with PCa death across cohorts, supporting the pooling of data from all cohorts.

The association between the TyG index (both as a linear term and in quartiles) and PCa incidence and death was modelled using Cox proportional hazards regression. To assess mediation of the effect of BMI on PCa death through the TyG index, we applied the two-stage regression method for survival data by VanderWeele [32]. In brief, two regression models were fit to the data, one modelling the mediator and the other modelling the outcome; parameter estimates of these two separate models were combined to obtain effect estimates of mediation. We modelled the outcome (PCa death) using Cox proportional hazards regression models, and the mediator (TyG index) using linear regression models. All these analyses were performed both on the full, initially cancer-free population, using attained age (left-truncated at the baseline examination) as the underlying time variable, and on PCa cases only, using time from PCa diagnosis to PCa-specific death as the time variable. Analogous mediation analyses using BMI categories instead of continuous BMI were performed. All models were stratified on cohort and birth decade (≤1929/1930-1939/1940-1949/1950-1959/1960-1969/ ≥ 1970), and adjusted for age at baseline (full cohort analysis), age at diagnosis (PCa-case only analysis), smoking status, and fasting status. In analyses of men included in the Swedish NPCR, further adjustments were made for country of birth, educational level, income, source of income, civil status, and Charlson comorbidity index [33] closest to the time of diagnosis, primary treatment for prostate cancer, and prostate cancer risk category. We found no interaction between BMI and TyG index on risk of PCa death; thus, no such interaction terms were added to the models.

**Fig. 1:**
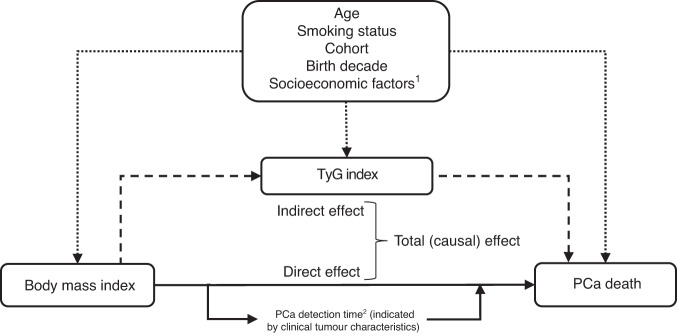
Directed acyclic graph (DAG) illustrating the conceptual framework and causal relationships between body mass index, the TyG index, and death from prostate cancer, including variables accounted for in our statistical models to reduce potential bias. Dotted arrows represent confounding pathways, whereas the other arrows (solid [direct effect of body mass index] and dashed [indirect effect via mediator TyG index]), due to their unidirectionality, can convey exposure effects to the outcome, and are thus causal pathways. ^1^Only available for Swedish men. ^2^The case-only analysis of time from PCa diagnosis to PCa-specific death is sensitive to when the tumour was diagnosed (“PCa detection time”). Delayed detection of PCa is more likely in obese compared to normal weight men [1, 3, 40]. Therefore, for the case-only analysis, PCa detection time can be seen as a subcomponent of the direct effect. However, delayed detection of PCa in obese men can also be interpreted as an instance of detection bias. Assuming that clinical tumour characteristics are an indicator of the detection time of the PCa, the influence of this bias can be mitigated by adjusting for clinical tumour characteristics, as has been done in secondary analyses in Swedish NPCR cases, for whom this information was available. PCa – prostate cancer.

Assuming causal relationships between variables as shown in the directed acyclic graph in Fig. 1, and under the assumption that we accounted for the majority of confounding, VanderWeele’s method [32] decomposes the total effect of BMI on PCa death (expressed as the hazard ratio (HR) per 5-kg/m^2^ increase in BMI, or as the HR vs. the reference <25 kg/m^2^ for BMI categories) into two components: the natural indirect effect (i.e. the effect of BMI that is due to mediation through the TyG index), and the natural direct effect (i.e. the effect of BMI not explained through the mediator) [32]. Since the question of mediation is of an intrinsically causal nature, we used the term “effect” in this context [34], even though our analysis is based on observational data. The proportion of the total effect of BMI on PCa death mediated through the TyG index (in %) was calculated on the log-transformed HR scale as log(indirect effect HR)/log(total effect HR) × 100, since HRs are additive on this scale. 95% confidence intervals (CIs) for estimates of the total, natural indirect, and natural direct effects and the proportion mediated were calculated based on standard errors (SEs) derived from the delta method.

As parameter (β) estimates from regression models might be diluted by random measurement error and long-term intra-individual fluctuations, we calculated regression dilution ratios (RDRs) for BMI and the TyG index, and corrected β estimates of all regression models (including those from the mediation models) by dividing the original values by the RDR. Consequently, for HRs from Cox regression models, the correction formula was ${{HR}}_{{corrected}}=\exp (\log \left({{HR}}_{{original}}\right)/{RDR})$. RDRs were calculated using linear mixed effects models as described by Wood et al. using all available repeated measurements in our cohorts [35, 36]. Details about the obtained RDRs and a comparison of characteristics of men with vs. without repeated measurements are shown in Figure S2 and Table S1, respectively.

RDRs were calculated using the user-written function *“rdrcalc”* (see https://www.phpc.cam.ac.uk/ceu/erfc/programmes/) using Stata SE, version 17.0. All other analyses were conducted in R, version 4.0.5 (R Foundation).

## Results

### Study population

Out of 259,884 study participants, 111,168 (42.8%) men originated from Norway, 80,098 (30.8%) from Austria, and 68,618 (26.4%) from Sweden. The mean age at baseline was 43.3 [standard deviation (SD) 10.1] years. More than half of the population was either overweight (43.6%) or obese (10.9%). Mean (SD) values of glucose (mmol/l), triglycerides (mmol/l) and the TyG index {ln[mg^2^/(2*dl^2^)]} were 5.3 (1.3), 1.8 (1.3), and 8.8 (0.6), respectively. Over a median follow-up time of 17.5 years, equalling a total of 5,150,512 person-years, 11,760 men were diagnosed with PCa, of whom 1784 died from the disease (Table 1**;** Table S2 for stratification by country). Among the 4101 Swedish PCa cases, 3820 (93.1%) were identified in the NPCR. Clinical characteristics for men in the NPCR are presented in Table S3.

**Table 1:** Characteristics of the 259,884 men in the study, overall, and stratified by quartiles of TyG index^a^.

	TyG index				Total (*N* = 259,884)
	Quartile 1^a^ (*N* = 65,013)	Quartile 2^a^ (*N* = 64,960)	Quartile 3^a^ (*N* = 64,951)	Quartile 4^a^ (*N* = 64,960)	
Cohort (year of baseline examination), *n* (%)					
Swedish cohorts					
VIP (1986–2016)	-^b^	-	-	-	51,993 (20.0%)
MONICA (1986–2004)	-	-	-	-	1001 (0.4%)
MDCS (1991–1995)	-	-	-	-	2213 (0.9%)
MPP (1978–2006)	-	-	-	-	13,411 (5.2%)
Norwegian cohorts					
Oslo study I (1972–1973)	-	-	-	-	17,471 (6.7%)
NCS (1974–1983)	-	-	-	-	30,804 (11.9%)
40-y (1993–1999)	-	-	-	-	62,893 (24.2%)
Austrian cohort					
VHM&PP (1988–2005)	-	-	-	-	80,098 (30.8%)
Birth year, median (Q1, Q3)	1954 (1940, 1959)	1953 (1937, 1958)	1952 (1937, 1957)	1950 (1936, 1956)	1953 (1937, 1958)
Age at baseline [years], mean (SD)	41.3 (10.2)	43.1 (10.3)	44.0 (10.0)	44.9 (9.5)	43.3 (10.1)
Smoking status, *n* (%)					
Never smoker	31,600 (48.6%)	28,541 (43.9%)	26,499 (40.8%)	23,880 (36.8%)	110,520 (42.5%)
Ex-smoker	17,161 (26.4%)	17,796 (27.4%)	18,451 (28.4%)	19,165 (29.5%)	72,573 (27.9%)
Current smoker	15,825 (24.3%)	18,227 (28.1%)	19,611 (30.2%)	21,462 (33.0%)	75,125 (28.9%)
Missing	427 (0.7%)	396 (0.6%)	390 (0.6%)	453 (0.7%)	1666 (0.6%)
Body mass index [kg/m^2^], mean (SD)	24.2 (2.9)	25.2 (3.2)	26.1 (3.4)	27.5 (3.8)	25.7 (3.5)
Body mass index [kg/m^2^], *n* (%)					
Normal weight (<24.9 kg/m^2^)	42,631 (65.6%)	33,752 (52.0%)	25,706 (39.6%)	16,384 (25.2%)	118,473 (45.6%)
Overweight (25 to 29.9 kg/m^2^)	20,195 (31.1%)	26,741 (41.2%)	31,598 (48.6%)	34,672 (53.4%)	113,206 (43.6%)
Obese (≥30.0 kg/m^2^)	2,187 (3.4%)	4467 (6.9%)	7647 (11.8%)	13,904 (21.4%)	28,205 (10.9%)
Fasting status, *n* (%)					
Less than 8 h	-^b^	-	-	-	111,343 (42.8%)
8 h or more	-	-	-	-	148,541 (57.2%)
Glucose [mmol/L], mean (SD)	4.9 (0.8)	5.2 (0.8)	5.4 (1.0)	6.0 (2.0)	5.3 (1.3)
Fasting (≥8 h) samples only	4.7 (0.8)	5.0 (0.8)	5.2 (0.9)	5.9 (2.0)	5.2 (1.3)
Triglycerides [mmol/L], mean (SD)	0.9 (0.2)	1.3 (0.3)	1.8 (0.4)	3.3 (1.7)	1.8 (1.3)
Fasting (≥8 h) samples only	0.8 (0.2)	1.1 (0.2)	1.6 (0.3)	3.0 (1.7)	1.6 (1.2)
TyG index^c^	8.1 (0.3)	8.5 (0.2)	8.9 (0.2)	9.5 (0.4)	8.8 (0.6)
Fasting (≥8 h) samples only	7.9 (0.3)	8.4 (0.1)	8.8 (0.1)	9.4 (0.4)	8.6 (0.6)
Follow-up [years], median (Q1, Q3)	17.7 (14.5, 25.6)	17.5 (13.8, 25.4)	17.5 (13.5, 25.2)	17.5 (13.5, 24.7)	17.5 (13.6, 25.2)
PCa diagnosis during F/U, *n* (%)	2747 (4.2%)	3009 (4.6%)	3065 (4.7%)	2939 (4.5%)	11,760 (4.5%)
Age at date of PCa diagnosis [years], mean (SD)	68.1 (8.5)	68.1 (8.4)	68.3 (8.3)	67.8 (8.1)	68.0 (8.3)
PCa deaths after diagnosis, *n* (%)^d^	388 (14.1%)	443 (14.7%)	496 (16.2%)	457 (15.5%)	1784 (15.2%)
F/U after date of PCa diagnosis [years], median (Q1, Q3)	5.8 (2.7, 10.0)	6.1 (2.7, 10.2)	5.9 (2.5, 10.0)	5.6 (2.5, 9.6)	5.8 (2.6, 9.9)

*VIP* Västerbotten Intervention Programme, *MONICA* Northern Sweden Monica Study, *MDCS* Malmö Diet and Cancer Study, *MPP* Malmö Preventive Project, *NCS* Norwegian Counties Study, *40-y* 40-year programme, *VHM&PP* Vorarlberg Health Monitoring and Prevention Programme, *F/U* follow-up, *PCa* prostate cancer, *SD* standard deviation.

^a^z-transformed TyG index values, transformation performed separately for cohort and fasting status (<8 h vs. ≥8 h).

^b^Due to the stratification of the z-transformation of the TyG index by cohort and fasting status, these proportions are the same as in the Total column.

^c^TyG index calculated as ln[triglycerides (mg/dL) × blood glucose (mg/dL)/2].

^d^Percentages are based on the total number of PCa diagnoses.

### Associations of TyG index with baseline characteristics and death from PCa

Mean baseline BMI and age, and the prevalence of smoking, increased across TyG index quartiles, while the number of PCa diagnoses was similar across quartiles (Table 1). The correlation of BMI with the TyG index remained after adjusting for cohort, birth decade, age, smoking, and fasting status (partial Pearson correlation *ρ* = 0.35).

In case-only analyses, the TyG index was positively associated with time from PCa diagnosis to PCa-specific death (HR_1-SD increase in TyG index_ = 1.11, 95% CI: 1.01–1.22) (Table 2). Analysing the TyG index categorised into quartiles confirmed a roughly linear relationship. The association became more pronounced when restricting the analysis to PCa cases with blood drawn in the fasting state (HR = 1.27, 95% CI: 1.12–1.44). The results did not change substantially when assessing only TyG index values measured at most 10 years prior to PCa diagnosis (HR = 1.15, 95% CI: 0.99–1.33), and when additionally adjusting for PCa risk category and other clinical characteristics in the NPCR cases.

**Table 2:** Hazard ratios of death from PCa by TyG index^a^.

	Model^b^	PCa deaths/N	TyG index^a^, HR (95% CI)^c^				HR of TyG index^c,d^ (per 1-SD increase)
			Quartile 1 (Ref)	Quartile 2	Quartile 3	Quartile 4	
**PCa-case only analysis (time from date of diagnosis)**							
All cases	Model 1	1784/11,760	1.00 (Ref)	1.03 (0.90–1.18)	1.15 (1.00–1.32)	1.17 (1.01–1.35)	1.11 (1.01–1.22)
Cases with fasting samples	Model 1	958/7794	1.00 (Ref)	1.16 (0.95–1.41)	1.34 (1.10–1.62)	1.38 (1.13–1.69)	1.27 (1.12–1.44)
Cases diagnosed with PCa within 10 years after baseline^e^	Model 1	695/5688	1.00 (Ref)	1.22 (0.97–1.53)	1.27 (1.01–1.59)	1.23 (0.97–1.57)	1.15 (0.99–1.33)
Swedish cases in the NPCR	Model 1	453/3820	1.00 (Ref)	1.07 (0.81–1.42)	1.38 (1.05–1.82)	1.30 (0.97–1.74)	1.23 (1.02–1.48)
Swedish cases in the NPCR	Model 2	453/3820	1.00 (Ref)	1.04 (0.78–1.39)	1.40 (1.06–1.86)	1.33 (0.99–1.78)	1.24 (1.02–1.52)
**Full cohort analysis (age at PCa-specific death)**							
All men	Model 3	1784/259,884	1.00 (Ref)	0.99 (0.86–1.13)	1.05 (0.92–1.21)	1.00 (0.87–1.15)	1.03 (0.94–1.13)
Men with fasting samples	Model 3	958/148,541	1.00 (Ref)	1.06 (0.87–1.29)	1.21 (1.00–1.46)	1.11 (0.91–1.36)	1.13 (1.00–1.29)
Swedish cohorts with cases in the NPCR	Model 3	453/68,337	1.00 (Ref)	1.08 (0.81–1.43)	1.32 (1.01–1.74)	1.21 (0.90–1.61)	1.16 (0.97–1.40)

*CI* confidence interval, *HR* hazard ratio, *NPCR* National Prostate Cancer Register, *PCa* prostate cancer, *SD* standard deviation.

^a^z-transformed TyG index values, transformation performed separately for cohort and fasting status (<8 h vs. ≥8 h).

^b^Model 1: adjusted for age at PCa diagnosis, body mass index, smoking status, fasting status, and stratified on cohort and birth decade. Model 2: adjusted for the same variables as in Model 1, plus additionally adjusted for country of birth, education at the time of diagnosis, income closest to diagnosis (categorical), source of income closest to diagnosis, civil status closest to diagnosis, Charlson comorbidity index, primary treatment for prostate cancer, and prostate cancer risk category. Model 3: adjusted for age at study entry, body mass index, smoking status, fasting status, and stratified on cohort and birth decade.

^c^HRs for TyG index were estimated in Cox proportional hazards models with attained age as the underlying time scale (beginning at the time of baseline examination) for the full cohort analysis, and time since PCa diagnosis as the underlying time scale for the PCa-case only analysis.

^d^HRs for TyG index as a continuous variable were corrected for the regression dilution ratio of 0.548. Conversion into the uncorrected hazard ratios can be obtained using the equation ${{HR}}_{{original}}=\exp (\log \left({{HR}}_{{corrected}}\right)\times 0.548)$.

^e^In case that the baseline visit occurred more than 10 years prior to PCa diagnosis, the first follow-up examination falling within the 10 year time window before PCa diagnosis, if available, was used.

In contrast, in the full cohort analysis the association between TyG index and PCa-specific death was much weaker (HR_1-SD increase in TyG index_ = 1.03, 95% CI: 0.94–1.13), although exclusion of non-fasting samples made the association stronger (HR = 1.13, 95% CI: 1.00–1.29) (Table 2). Notably, the TyG index was slightly negatively associated with PCa incidence (0.96, 95% CI: 0.93–1.00).

### Effect of BMI on death from PCa mediated through the TyG index

Analysing time since PCa diagnosis in PCa cases only, BMI was positively associated with the risk of PCa death (HR_total effect_ = 1.24 per 5-kg/m^2^ increase in BMI, 95% CI: 1.14–1.35). Of this total effect, 28% (95% CI: 4%–52%) were mediated through the TyG index (HR_indirect effect_ = 1.06, 95% CI: 1.01–1.12) (Table 3; Table S4 for RDR-uncorrected results). The proportion increased to 54% (95% CI: 22%–85%) when analysing fasting blood samples only. Analysis by BMI categories yielded a total effect HR of 1.13 (95% CI: 1.02–1.25) for overweight vs. normal weight, and a total effect HR of 1.59 (95% CI: 1.34–1.89) for obese men. Proportions mediated through the TyG index were 42% for overweight (HR_indirect effect_ = 1.05; 95% CI: 1.01–1.10), and 22% for obesity (HR_indirect effect_ = 1.11; 95% CI: 1.02–1.21). Assessing only BMI and TyG index values measured at most 10 years prior to PCa diagnosis, and further adjustment by PCa risk category and other clinical characteristics for Swedish men in the NPCR did not materially change the effects (Table 3).

**Table 3:** PCa-case only analysis of the effect of BMI on time from PCa diagnosis to PCa death mediated through the TyG index.

	Model^a^	BMI, kg/m^2^	PCa deaths/N	Total effect HR (95% CI)^b^	Natural direct effect HR (95% CI)^b^	Natural indirect effect HR (95% CI)^b^	Proportion mediated % (95% CI)
All cases	Model 1	<25	817/5361	1.00 (Reference)	-	-	-
		25 ≤ 30	789/5377	1.13 (1.02–1.25)	1.08 (0.97–1.19)	1.05 (1.01–1.10)	42% (−1%–85%)
		≥30	178/1022	1.59 (1.34–1.89)	1.44 (1.21–1.70)	1.11 (1.02–1.21)	22% (4%–40%)
		Per 5	1784/11,760	1.24 (1.14–1.35)	1.17 (1.07–1.27)	1.06 (1.01–1.12)	28% (4%–52%)
Cases with fasting samples	Model 1	<25	369/3259	1.00 (Reference)	-	-	-
		25 ≤ 30	460/3724	1.22 (1.05–1.40)	1.09 (0.94–1.26)	1.12 (1.05–1.19)	57% (10%–104%)
		≥30	129/811	1.75 (1.41–2.16)	1.40 (1.13–1.74)	1.24 (1.10–1.40)	39% (17%–61%)
		Per 5	958/7794	1.29 (1.15–1.44)	1.13 (1.00–1.26)	1.15 (1.07–1.23)	54% (22%–85%)
Cases diagnosed with PCa within 10 years after baseline^c^	Model 1	<25	244/2034	1.00 (Reference)	-	-	-
		25 ≤ 30	327/2852	1.09 (0.91–1.29)	1.02 (0.86–1.21)	1.07 (1.00–1.14)	78% (−90%–246%)
		≥30	124/802	1.69 (1.34–2.12)	1.48 (1.18–1.87)	1.14 (0.99–1.30)	25% (0%–50%)
		Per 5	695/5688	1.26 (1.11–1.43)	1.17 (1.03–1.33)	1.08 (1.00–1.17)	33% (−1%–8%)
Swedish cases in the NPCR	Model 1	<25	188/1637	1.00 (Reference)	-	-	-
		25 ≤ 30	217/1814	1.18 (0.96–1.45)	1.07 (0.87–1.31)	1.10 (1.00–1.21)	59% (−24%–142%)
		≥30	48/369	1.42 (1.01–1.98)	1.17 (0.84–1.64)	1.21 (1.01–1.44)	54% (−8%–116%)
		Per 5	453/3820	1.17 (0.99–1.39)	1.04 (0.87–1.23)	1.13 (1.01–1.26)	77% (−12%–167%)
Swedish cases in the NPCR	Model 2	<25	188/1637	1.00 (Reference)	-	-	-
		25 ≤ 30	217/1814	1.16 (0.93–1.43)	1.06 (0.86–1.31)	1.09 (1.00–1.19)	60% (−37%–157%)
		≥30	48/369	1.58 (1.10–2.25)	1.35 (0.94–1.93)	1.17 (0.99–1.38)	35% (−5%–75%)
		Per 5	453/3820	1.17 (0.98–1.39)	1.04 (0.88–1.25)	1.12 (1.01–1.24)	72% (−17%–161%)

*BMI* body mass index, *CI* confidence interval, *HR* hazard ratio, *NPCR* National Prostate Cancer Register, *PCa* prostate cancer.

^a^Model 1: adjusted for age at PCa diagnosis, body mass index, smoking status, fasting status, and stratified on cohort and birth decade. Model 2: adjusted for the same variables as in Model 1, plus additionally adjusted for country of birth, education at the time of diagnosis, income closest to diagnosis, source of income closest to diagnosis, civil status closest to diagnosis, Charlson comorbidity index, primary treatment for prostate cancer, and prostate cancer risk category.

^b^HRs were estimated according to the two-stage regression method proposed by VanderWeele, with time since PCa diagnosis as the underlying time scale. 95% confidence intervals (CIs) were computed using the Delta method. All estimated effects of the mediation models were corrected for the regression dilution ratio of 0.924 for BMI (only when used as a continuous variable) and 0.548 for the TyG index, by dividing all β estimates of the mediator and outcome model by the respective RDR. To assess the impact of this correction, we are also providing the results of mediation analyses not correcting for regression dilution ratios in Table S4.

^c^In case that the baseline visit occurred more than 10 years prior to PCa diagnosis, the first follow-up examination falling within the 10 year time window before PCa diagnosis, if available, was used.

Analysing age at PCa death in the full cohort, associations of BMI with PCa death were slightly weaker than in the case-only analyses (HR_total effect_ = 1.17, 95% CI: 1.08–1.27). Only 11% (95% CI: −21%–42%) of this total effect [38% (95% CI: −1%–77%) for fasting samples only], and thus a markedly smaller proportion than for the PCa-case only analysis, were mediated through the TyG index (HR_indirect effect, total_ = 1.02, 95% CI: 0.97–1.07; HR_indirect effect, fasting samples only_ = 1.07; 95% CI: 1.00–1.14) (Table 4).

**Table 4:** Full cohort analysis of the effect of BMI on time from baseline examination to PCa death mediated through the TyG index.

	BMI, kg/m^2^	PCa deaths/N	Total effect HR (95% CI)^a^	Natural direct effect HR (95% CI)^a^	Natural indirect effect HR (95% CI)^a^	Proportion mediated % (95% CI)
All men	<25	817/118,473	1.00 (Reference)	-	-	-
	25 ≤ 30	789/113,206	1.11 (1.00–1.23)	1.09 (0.98–1.20)	1.02 (0.97–1.07)	18% (−27%–64%)
	≥30	178/28,205	1.43 (1.21–1.71)	1.38 (1.16–1.64)	1.04 (0.95–1.14)	10% (−14%–35%)
	Per 5	1784/259,884	1.17 (1.08–1.27)	1.15 (1.06–1.25)	1.02 (0.97–1.07)	11% (−21%–42%)
Men with fasting samples	<25	369/66,727	1.00 (Reference)	-	-	-
	25 ≤ 30	460/63,563	1.18 (1.02–1.36)	1.11 (0.97–1.29)	1.06 (0.99–1.13)	35% (−8%–79%)
	≥30	129/18,251	1.52 (1.23–1.88)	1.36 (1.10–1.68)	1.12 (0.99–1.26)	27% (−2%–55%)
	Per 5	958/148,541	1.19 (1.07–1.32)	1.11 (1.00–1.24)	1.07 (1.00–1.14)	38% (−1%–77%)
Swedish cohorts with cases in the NPCR	<25	188/27,911	1.00 (Reference)	-	-	-
	25 ≤ 30	217/31,008	1.18 (0.97–1.45)	1.10 (0.90–1.35)	1.07 (0.98–1.18)	42% (−25%–109%)
	≥30	48/9,418	1.28 (0.91–1.79)	1.12 (0.80–1.56)	1.15 (0.96–1.38)	56% (−34%–146%)
	Per 5	453/68,337	1.13 (0.96–1.32)	1.04 (0.88–1.22)	1.08 (0.98–1.20)	69% (−38%–176%)

*BMI* body mass index, *CI* confidence interval, *HR* hazard ratio, *NPCR* National Prostate Cancer Register, *PCa* prostate cancer.

^a^HRs were estimated according to the two-stage regression method proposed by VanderWeele, with attained age as the underlying time scale. All models were adjusted for age at study entry, smoking status, fasting status, and stratified on cohort and birth decade. 95% confidence intervals (CIs) were computed according to the Delta method. All estimated effects of the mediation models were corrected for the regression dilution ratio of 0.924 for BMI (only when used as a continuous variable) and 0.548 for the TyG index, by dividing all β estimates of the mediator and outcome model by the respective RDR. To assess the impact of this correction, we are also providing the results of mediation analyses not correcting for regression dilution ratios in Table S4.

## Discussion

In this large, pooled analysis of eight population-based European cohorts, higher TyG index was associated with a shorter time from PCa diagnosis to PCa-specific death, and more than a quarter of the total effect of BMI on PCa-specific death was mediated through the TyG index. In the full cohort followed from a cancer-free state through PCa diagnosis and death, there was neither an association of the TyG index with PCa-specific death, nor did the TyG index substantially mediate the effect of BMI on PCa death. These results suggest that insulin resistance, as reflected by the TyG index, may substantially mediate the effect of BMI on PCa progression to death, but not a diagnosis of PCa.

The different findings in the analysis of PCa cases who were followed from time of diagnosis as compared to the analysis of the full cohort followed from baseline throughout the follow-up period, both commonly applied approaches, warrant further discussion. The results from the full cohort analysis reflect the association of obesity with a mix of time until PCa diagnosis and PCa-specific death. The second component is of primary interest in the study of PCa progression and survival, and was directly studied in our case-only analysis. Importantly, obesity and insulin resistance are hypothesised to be more involved during the progression of PCa [1, 17, 20]. Our finding that the proportion of the effect of baseline BMI on PCa death mediated through the TyG index is more pronounced in the case-only analysis compared to the full cohort analysis supports this hypothesis. This interpretation was further corroborated by the analysis of data measured up to ten years prior to PCa diagnosis. However, the relationship of BMI and TyG index measured at the time of PCa diagnosis, together with changes from baseline, with PCa-specific death should be investigated in future studies.

While time from diagnosis to PCa-specific death was studied directly in the case-only analysis, this analysis might be affected by collider stratification bias inherent to the analysis of cases only [37–39]. However, for collider bias to influence our findings, an association between TyG index and incident PCa would be required and this association was weak in our data. The case-only analysis might also be affected by detection bias resulting in a differential exposure association with indolent and advanced PCa at diagnosis, which has been observed for BMI [1, 3, 40]. Delayed diagnosis of PCa is more likely in obese compared to normal weight men because of hemodiluted prostate-specific antigen (PSA) levels and enlarged prostate glands, and possibly lower frequency of asymptomatic PSA testing in obese men [1, 3, 40]. In analyses of cases in the NPCR of Sweden, we were able to mitigate the potential influence of differential detection time by adjusting the statistical models for PCa risk category and other clinical characteristics. However, these additional adjustments did not materially change the results, which validates the unadjusted results of our extended pooling.

In addition to epidemiological studies, which have shown that insulin resistance is associated with PCa progression and PCa-specific death [26, 27], there is also evidence from preclinical and mechanistic studies of the importance of insulin resistance in PCa progression. For example, increased numbers of insulin receptors on the cell membranes of high grade compared to low grade prostate tumours have been observed [41]. Another study reported an increased expression of the insulin receptor A isoform in high grade PCa cells in vitro and in vivo [42]. Diet-induced hyperinsulinemia was associated with PCa tumour growth in a xenograft model [43]. Finally, a relatively recent study reported that insulin made PCa cells in vitro more invasive and mobile [44]. However, a large proportion of the BMI effect was not mediated through the TyG index in our study. Although unaccounted measurement error and confounding might have led to an underestimation of the indirect effect, other important mechanisms are likely to be in action. Other mechanisms for which the presence of mediation should be studied as well include (i) alterations in sex hormone metabolism, in particular androgen deficiency [1, 45, 46], (ii) chronic inflammation characterised by altered levels of adipokines in obese men [17, 47, 48], and (iii) less successful treatment in obese men with associated higher rates of disease recurrence [1, 18, 19]. In general, proportions mediated through the TyG index appeared to be larger for overweight than for obesity in our analyses. This observation might be explained by the sometimes expressed claim that overweight per se is not as intrinsically bad as obesity, and exerts its detrimental effects to a higher extent via metabolic pathways, compared to obesity, where other pathways, such as neurohumoral or hemodynamic dysregulations, are additional relevant contributors [49, 50].

One limitation of our study was that we only had measures of BMI and TyG index. Those are only surrogate measures of excess body fatness and insulin resistance, the conditions that are believed to be the biological link to PCa death. Furthermore, the precision of the TyG index may be reduced when measured in the non-fasting state. This would dilute the association and explain why the proportion of the effect of BMI on PCa death risk mediated through the TyG index was markedly smaller when analysing all men compared to analysing only samples from fasting men. However, systematic differences across cohorts might be another reason, since men with non-fasting samples originated primarily from the Norwegian cohorts. Furthermore, BMI and the TyG index were measured at the same time in point, while ideally the exposure BMI should be measured prior to the mediator TyG index. However, BMI is a very stable measure over the observation period of our study population (RDR = 0.924), so the simultaneous measurement of BMI and TyG index probably does not substantially affect our findings, in particular because we corrected our analyses for the RDR. Another direct limitation of observational research is that we cannot rule out the possibility of unmeasured and/or residual confounding.

Strengths of our study include the large sample size and long follow-up, the use of high-quality national cancer registers ensuring a virtually complete capture of cancer cases [51–53], detailed and validated cancer characteristics for a large proportion of the Swedish PCa cases [54], and the availability of repeated measurements to account for random measurement error and intra-individual fluctuations in BMI and TyG index values. Notably, mediation analysis corrected for the RDR (Tables 3, 4) showed that the proportion of the effect of BMI on PCa death risk mediated through the TyG index was underestimated by 10% to 15% in uncorrected analysis (Table S4), in line with previous studies [35, 36, 55].

In summary, in PCa-case only analyses the TyG index was positively associated with PCa-specific death, and more than a quarter of the effect of BMI on PCa death was mediated through the TyG index. The contribution of the TyG index as a mediator to the effect of BMI on PCa-specific death in the full cohort was much smaller, because, in contrast to PCa death, the TyG index was slightly negatively associated with PCa incidence. As the TyG index is indicative of insulin resistance, our findings support a role for insulin in promoting PCa progression. Our results provide further evidence of the importance of avoiding excess weight and maintaining a healthy metabolic profile, and adds to the rationale for investigating novel treatment strategies for PCa targeting insulin resistance as an adjuvant therapy for PCa.

### Supplementary information

Supplemental_Information_BMI_TyG_mediation_PCa_death

## Data Availability

The data that support the findings of this study are available from cohort committees and national registers of the cohorts and countries involved. Restrictions apply to the availability of these data, which were used under license for this study. Data are available after contact with the corresponding author conditional on permission from the involved cohort committees and national registers.
