# Supplementary material for: Body mass index, triglyceride-glucose index, and prostate cancer death: a mediation analysis in eight European cohorts
Source: Br J Cancer. 2023 Dec 12;130(2):308–16. doi: 10.1038/s41416-023-02526-1 (PMC10803806; doi:10.1038/s41416-023-02526-1)
Supplement: Supplementary file 1 — Supplemental_Information_BMI_TyG_mediation_PCa_death [file 41416_2023_2526_MOESM1_ESM.docx]

**Supplementary Information**

**Body mass index, triglyceride-glucose index, and prostate cancer death: a mediation analysis** **in eight European cohorts**

Josef Fritz, Sylvia H. J. Jochems, Tone Bjørge, Angela M. Wood, Christel Häggström, Hanno Ulmer, Gabriele Nagel, Emanuel Zitt, Anders Engeland, Sophia Harlid, Isabel Drake, Pär Stattin, Tanja Stocks

Corresponding author: Josef Fritz

Email: [josef.fritz@med.lu.se](file:///C:\Users\CASEL\AppData\Roaming\Microsoft\Word\josef.fritz@med.lu.se)

[Supplementary Materials and Methods 2](#_Toc129366403)

[References 4](#_Toc129366404)

[Table S1: Characteristics of the 259,884 men in the study, with vs. without repeated measurements of body mass index and the TyG index. 7](#_Toc129366405)

[Table S2: Characteristics of the 259,884 men in the study, overall, and stratified by country. 9](#_Toc129366406)

[Table S3: Clinical characteristics of the 3,820 incident prostate cancer cases in the Swedish cohorts which are recorded in the Swedish National Prostate Cancer Register. 11](#_Toc129366407)

[Table S4. Mediation analysis of the effect of BMI on PCa death without correction for regression dilution ratio. 13](#_Toc129366408)

[Figure S1. Flowchart showing the exclusion and selection of men and observations (health examinations) in the study. 14](#_Toc129366409)

[Figure S2. Overall and cohort and time-specific regression dilution ratios (95% confidence intervals) of (A) body mass index and (B) the TyG index1. 15](#_Toc129366410)

Supplementary Materials and Methods

**Cohorts**

The study included data from the following eight population-based cohorts: (1) the Västerbotten Intervention Programme (VIP) [northern Sweden]^1,2^, (2) the Northern Sweden Monica (MONICA) study [northern Sweden]^3,4^, (3) the Malmö Diet and Cancer Study (MDCS) [southern Sweden]^5,6^, (4) the Malmö Preventive Project (MPP) [southern Sweden]^7,8^, (5) the Oslo study I [Norway]^9,10^, (6) the Norwegian Counties Study (NCS) [Norway]^11,12^, (7) the 40-year programme (40-y) [Norway]^13^, and (8) the Vorarlberg Health Monitoring and Prevention Programme (VHM&PP) [Austria]^14,15^. Data from health examinations in these cohorts were included for the period 1986-2016 in VIP, 1986-2004 in MONICA, 1991-1995 in MDCS, 1978-2006 in MPP, 1972-1973 in the Oslo study I, 1974-1983 in NCS, 1993-1999 in 40-y, and 1988-2005 in VHM&PP. Further cohort details can be found in the respective references. The study was approved by research ethics committees in the respective countries (Norway: Regional Committee for Medical and Health Research Ethics, no 2012/2271/REC South-East, Sweden: EPN Umeå, no 2012-354-31M and no 2015-7-32M, and EPN Lund, no 2016/564 and 2020-01571, Austria: Ethics Committee of the province of Vorarlberg, no 2006-6/2).

**Additional register linkages in Sweden**

For the four Swedish cohorts, additional information on socioeconomic factors and country of birth were obtained from the Longitudinal Integration Database for Health Insurance and Labour Market Studies (LISA)^16^, and information on in-patient care (which was used for deriving the Charlson comorbidity index (CCI)) from the Patient Register^17^. Clinical information of PCa cases (*e.g.* tumour characteristics at PCa diagnosis, primary treatment) was obtained via linkage to the National Prostate Cancer Register (NPCR), which became nationwide in 1998 and has captured more than 99% of all PCa cases^18,19^. Between 1987 and 1998, the NPCR also included information from regional PCa registers. PCa's were classified into five risk groups: (i) localised low-risk (T1-2, Gleason score 2-6, and prostate-specific antigen [PSA] level <10 ng/mL), (ii) localised intermediate-risk (T1-2, Gleason score 7, and/or PSA 10 to <20 ng/mL), (iii) localised high-risk (T3, and/or Gleason score 8-10, and/or PSA 20 to <50 ng/mL), (iv) regionally metastatic/locally advanced (T4, and/or N1, and/or PSA 50 to <100 ng/mL in the absence of distant metastases [M0 or Mx]), and (v) distant metastases (M1 and/or PSA ≥100 ng/mL)^18^.

**Exclusions**

Out of a total of 774,029 health examinations in 431,525 men, we excluded duplicates, men with inconsistent height measurements, inconsistencies in cancer and death dates, and men with PCa as the documented cause of death but without a PCa diagnosis recorded in the respective Cancer Register. We also excluded visits performed before the age of 21, and visits with missing or implausible information on BMI, fasting time before measurement, glucose and triglycerides. The majority of these exclusions were due to missing information on glucose levels in the Norwegian cohorts during years when it had not been routinely measured. The first of the remaining visits was defined as the baseline visit. After excluding men with a cancer diagnosis before the baseline visit (excluding non-melanoma skin cancer), 259,884 men with 488,833 health examinations were left for our final analysis population (**Figure S1**).

References

1. Weinehall L, Hallgren CG, Westman G, Janlert U, Wall S. Reduction of selection bias in primary prevention of cardiovascular disease through involvement of primary health care. *Scand J Prim Health Care*. 1998;16(3):171-176. doi:10.1080/028134398750003133

2. Lindahl B, Weinehall L, Asplund K, Hallmans G. Screening for impaired glucose tolerance: Results from a population- based study in 21,057 individuals. *Diabetes Care*. 1999;22(12):1988-1992. doi:10.2337/diacare.22.12.1988

3. Stegmayr B, Lundberg V, Asplund K. The events registration and survey procedures in the Northern Sweden MONICA Project. *Scand J Public Health*. 2003;31(61_suppl):9-17. doi:10.1080/14034950310001441

4. Eriksson M, Forslund AS, Jansson JH, Söderberg S, Wennberg M, Eliasson M. Greater decreases in cholesterol levels among individuals with high cardiovascular risk than among the general population: The northern Sweden MONICA study 1994 to 2014. *Eur Heart J*. 2016;37(25):1985-1992. doi:10.1093/eurheartj/ehw052

5. Berglund G, Elmstähl S, Janzon L, Larsson SA. The Malmo Diet and Cancer Study. Design and feasibility. *J Intern Med*. 1993;233(1):45-51. doi:10.1111/j.1365-2796.1993.tb00647.x

6. Smith J, Platonov P, Hedblad B, Engström G, Melander O. Atrial fibrillation in the Malmö Diet and Cancer study: a study of occurrence, risk factors and diagnostic validity. *Eur J Epidemiol*. 2010;25(2):95-102. doi:10.1007/s10654-009-9404-1

7. Berglund G, Eriksson KF, Israelsson B, et al. Cardiovascular risk groups and mortality in an urban Swedish male population: The Malmö Preventive Project. *J Intern Med*. 1996;239(6):489-497. doi:10.1046/j.1365-2796.1996.483819000.x

8. Berglund G, Nilsson P, Eriksson KF, et al. Long-term outcome of the Malmo Preventive Project: Mortality and cardiovascular morbidity. *J Intern Med*. 2000;247(1):19-29. doi:10.1046/j.1365-2796.2000.00568.x

9. Leren P, Askevold E, Foss O, et al. The Oslo study. Cardiovascular disease in middle-aged and young Oslo men. *Acta Med Scand Suppl*. 1975;588:1-38.

10. Håheim LL, Wisløff TF, Holme I, Nafstad P. Metabolic syndrome predicts prostate cancer in a cohort of middle-aged Norwegian men followed for 27 years. *Am J Epidemiol*. 2006;164(8):769-774. doi:10.1093/aje/kwj284

11. Bjartveit K, Foss O, Gjervig T. The cardiovascular disease study in Norwegian counties. Results from first screening. *Acta Med Scand Suppl*. 1983;675:1-184.

12. Tverdal A, Foss OP, Leren P, Holme I, Lund-larsen PG, Bjartveit K. Serum triglycerides as an independent risk factor for death from coronary heart disease in mtddle-aged norwegian men. *Am J Epidemiol*. 1989;129(3):458-465. doi:10.1093/oxfordjournals.aje.a115157

13. Aires N, Selmer R, Thelle D. The validity of self-reported leisure time physical activity, and its relationship to serum cholesterol, blood pressure and body mass index. A population based study of 332,182 men and women aged 40-42 years. *Eur J Epidemiol*. 2003;18(6):479-485. doi:10.1023/A:1024682523710

14. Ulmer H, Kelleher C, Diem G, Concin H. Long-term tracking of cardiovascular risk factors among men and women in a large population-based health system: The Vorarlberg Health Monitoring & Promotion Programme. *Eur Heart J*. 2003;24(11):1004-1013. doi:10.1016/S0195-668X(03)00170-2

15. Ulmer H, Kelleher C, Diem G, Concin H. Why Eve is not Adam: prospective follow-up in 149650 women and men of cholesterol and other risk factors related to cardiovascular and all-cause mortality. *J Womens Health (Larchmt)*. 2004;13(1):41-53. doi:10.1089/154099904322836447

16. Ludvigsson JF, Svedberg P, Olén O, Bruze G, Neovius M. The longitudinal integrated database for health insurance and labour market studies (LISA) and its use in medical research. *Eur J Epidemiol*. 2019;34(4). doi:10.1007/S10654-019-00511-8

17. Patientregistret [Internet]. Socialstyrelsen. [cited 2022 June 29]. Available from https://www.socialstyrelsen.se/statistik-och-data/register/alla-register/patientregistret/

18. Van Hemelrijck M, Wigertz A, Sandin F, et al. Cohort profile: The national prostate cancer register of sweden and prostate cancer data base Sweden 2.0. *Int J Epidemiol*. 2013;42(4):956-967. doi:10.1093/ije/dys068

19. Tomic K, Sandin F, Wigertz A, Robinson D, Lambe M, Stattin P. Evaluation of data quality in the National Prostate Cancer Register of Sweden. *Eur J Cancer*. 2015;51(1):101-111. doi:10.1016/j.ejca.2014.10.025

Table S1: Characteristics of the 259,884 men in the study, with vs. without repeated measurements of body mass index and the TyG index.

|  | **Men with only one examination**  **(N=173,753)** | **Men with more than one examination**  **(N=86,131)** |
| --- | --- | --- |
| **Cohort (year of baseline examination), n (%)** |  |  |
| **VIP (1986-2016)** | 33,087 (19.0%) | 18,906 (22.0%) |
| **MONICA (1986-2004)** | 662 (0.4%) | 339 (0.4%) |
| **MDCS (1991-1995)** | 2,213 (1.3%) | - |
| **MPP (1978-2006)** | 7,556 (4.3%) | 5,855 (6.8%) |
| **Oslo study I (1972-1973)** | 17,471 (10.1%) | - |
| **NCS (1974-1983)** | 22,914 (13.2%) | 7,890 (9.2%) |
| **40y (1993-1999)** | 62,863 (36.2%) | 30 (<0.1%) |
| **VHM&PP (1988-2005)** | 26,987 (15.5%) | 53,111 (61.7%) |
| **Number of examination, mean (SD) [min, max]** | 1.0 (0.0) [1, 1] | 3.7 (2.6) [2, 24] |
| **Average time between examination [years], mean (SD)** | - | 6.4 (5.9) |
| **Birth year, median (Q1, Q3)** | 1954 (1937, 1957) | 1949 (1938, 1959) |
| **Age at baseline [years], mean (SD)** | 43.5 (9.1) | 42.8 (11.9) |
| **Smoking status, n (%)** |  |  |
| **Never smoker** | 63,011 (36.3%) | 47,509 (55.2%) |
| **Ex-smoker** | 59,683 (34.3%) | 12,890 (15.0%) |
| **Current smoker** | 49,753 (28.6%) | 25,372 (29.5%) |
| **Missing** | 1,306 (0.8%) | 360 (0.4%) |
| **Body mass index [kg/m^2^], mean (SD)** | 25.9 (3.6) | 25.5 (3.4) |
| **Body mass index [kg/m^2^], n (%)** |  |  |
| **<25 kg/m^2^** | 76,838 (44.2%) | 41,635 (48.3%) |
| **25 to <30 kg/m^2^** | 76,985 (44.3%) | 36,221 (42.1%) |
| **≥30.0 kg/m^2^** | 19,930 (11.5%) | 8,275 (9.6%) |
| **Fasting status, n (%)** |  |  |
| **Less than 8h** | 100,954 (58.1%) | 10,389 (12.1%) |
| **8h or more** | 72,799 (41.9%) | 75,742 (87.9%) |
| **Glucose [mmol/L], mean (SD)** | 5.5 (1.3) | 5.1 (1.2) |
| **Fasting (≥8h) samples only** | 5.4 (1.3) | 5.0 (1.2) |
| **Triglycerides [mmol/L], mean (SD)** | 1.9 (1.3) | 1.7 (1.2) |
| **Fasting (≥8h) samples only** | 1.6 (1.2) | 1.7 (1.2) |
| **TyG index^1^** | 8.8 (0.6) | 8.6 (0.6) |
| **Fasting (≥8h) samples only** | 8.7 (0.6) | 8.6 (0.6) |
| **Follow-up [years], median (Q1, Q3)** | 16.5 (13.5, 23.2) | 22.7 (17.3, 25.9) |
| **PCa diagnosis during F/U, n (%)** | 6,462 (3.7%) | 5,298 (6.2%) |
| **Age at date of PCa diagnosis [years], mean (SD)** | 68.7 (8.2) | 67.2 (8.4) |
| **PCa deaths after diagnosis, n (%)^2^** | 1,218 (18.8%) | 566 (10.7%) |
| **Follow-up after date of PCa diagnosis [years], median (Q1, Q3)** | 4.9 (2.1, 8.8) | 7.1 (3.2, 10.9) |

^1^TyG index calculated as ln[triglycerides (mg/dL) × blood glucose (mg/dL)/2].

^2^Percentages are based on the total number of PCa diagnoses.

Abbreviations: VIP – Västerbotten Intervention Programme, MONICA – Northern Sweden Monica Study, MDCS – Malmö Diet and Cancer Study, MPP – Malmö Preventive Project, Oslo – Oslo study I, NCS – Norwegian Counties Study, 40-y – 40-year programme, VHM&PP – Vorarlberg Health Monitoring and Prevention Programme, F/U – follow-up, PCa – prostate cancer, SD – standard deviation.

Table S2: Characteristics of the 259,884 men in the study, overall, and stratified by country.

|  | **Swedish cohorts**  **(N=68,618)** | **Norwegian cohorts**  **(N=111,168)** | **Austrian cohort**  **(N=80,098)** | **Total**  **(N=259,884)** |
| --- | --- | --- | --- | --- |
| **Cohort (year of baseline examination), n (%)** |  |  |  |  |
| **VIP (1986-2016)** | 51,993 (75.8%) | - | - | 51,993 (20.0%) |
| **MONICA (1986-2004)** | 1,001 (1.5%) | - | - | 1,001 (0.4%) |
| **MDCS (1991-1995)** | 2,213 (3.2%) | - | - | 2,213 (0.9%) |
| **MPP (1978-2006)** | 13,411 (19.5%) | - | - | 13,411 (5.2%) |
| **Oslo study I (1972-1973)** | - | 17,471 (15.7%) | - | 17,471 (6.7%) |
| **NCS (1974-1983)** | - | 30,804 (27.7%) | - | 30,804 (11.9%) |
| **40y (1993-1999)** | - | 62,893 (56.6%) | - | 62,893 (24.2%) |
| **VHM&PP (1988-2005)** | - | - | 80,098 (100%) | 80,098 (30.8%) |
| **Birth year, median (Q1, Q3)** | 1950 (1940, 1962) | 1953 (1933, 1956) | 1953 (1940, 1964) | 1953 (1937, 1958) |
| **Age at baseline [years], mean (SD)** | 47.1 (9.3) | 41.5 (4.5) | 42.6 (14.5) | 43.3 (10.1) |
| **Smoking status, n (%)** |  |  |  |  |
| **Never smoker** | 37,856 (55.2%) | 22,973 (20.7%) | 49,691 (62.0%) | 110,520 (42.5%) |
| **Ex-smoker** | 14,437 (21.0%) | 49,676 (44.7%) | 8,460 (10.6%) | 72,573 (27.9%) |
| **Current smoker** | 15,229 (22.2%) | 37,949 (34.1%) | 21,947 (27.4%) | 75,125 (28.9%) |
| **Missing** | 1,096 (1.6%) | 570 (0.5%) | 0 (0%) | 1,666 (0.6%) |
| **Body mass index [kg/m^2^], mean (SD)** | 26.2 (3.8) | 25.6 (3.3) | 25.5 (3.7) | 25.7 (3.5) |
| **Body mass index [kg/m^2^], n (%)** |  |  |  |  |
| **<25 kg/m^2^** | 28,042 (40.9%) | 51,379 (46.2%) | 39,052 (48.8%) | 118,473 (45.6%) |
| **25 to <30 kg/m^2^** | 31,119 (45.4%) | 49,692 (44.7%) | 32,395 (40.4%) | 113,206 (43.6%) |
| **≥30.0 kg/m^2^** | 9,457 (13.8%) | 10,097 (9.1%) | 8,651 (10.8%) | 28,205 (10.9%) |
| **Fasting status, n (%)** |  |  |  |  |
| **Less than 8h** | 5,136 (7.5%) | 106,207 (95.5%) | 0 (0%) | 111,343 (42.8%) |
| **8h or more** | 63,482 (92.5%) | 4,961 (4.5%) | 80,098 (100%) | 148,541 (57.2%) |
| **Glucose [mmol/L], mean (SD)** | 5.4 (1.2) | 5.5 (1.3) | 5.0 (1.4) | 5.3 (1.3) |
| **Fasting (≥8h) samples only** | 5.4 (1.1) | 5.5 (1.0) | 5.0 (1.4) | 5.2 (1.3) |
| **Triglycerides [mmol/L], mean (SD)** | 1.5 (1.0) | 2.1 (1.3) | 1.7 (1.4) | 1.8 (1.3) |
| **Fasting (≥8h) samples only** | 1.5 (1.0) | 1.7 (1.3) | 1.7 (1.4) | 1.6 (1.2) |
| **TyG index^1^** | 8.6 (0.5) | 9.0 (0.6) | 8.6 (0.7) | 8.8 (0.6) |
| **Fasting (≥8h) samples only** | 8.6 (0.5) | 8.7 (0.6) | 8.6 (0.7) | 8.6 (0.6) |
| **Follow-up [years], median (Q1, Q3)** | 15.9 (8.9, 23.0) | 17.5 (14.5, 35.0) | 20.1 (14.0, 24.6) | 17.5 (13.6, 25.2) |
| **PCa diagnosis during F/U, n (%)** | 4,101 (6.0%) | 3,692 (3.3%) | 3,967 (5.0%) | 11,760 (4.5%) |
| **Age at date of PCa diagnosis [years], mean (SD)** | 68.1 (7.2) | 69.0 (8.6) | 67.2 (9.0) | 68.0 (8.3) |
| **PCa deaths after diagnosis, n (%)^2^** | 533 (13.0%) | 799 (21.6%) | 452 (11.4%) | 1,784 (15.2%) |
| **Follow-up after date of PCa diagnosis [years], median (Q1, Q3)** | 5.5 (2.3, 9.7) | 4.4 (2.0, 7.9) | 7.8 (3.8, 11.2) | 5.8 (2.6, 9.9) |

^1^TyG index calculated as ln[triglycerides (mg/dL) × blood glucose (mg/dL)/2].

^2^Percentages are based on the total number of PCa diagnoses.

Abbreviations: VIP – Västerbotten Intervention Programme, MONICA – Northern Sweden Monica Study, MDCS – Malmö Diet and Cancer Study, MPP – Malmö Preventive Project, Oslo – Oslo study I, NCS – Norwegian Counties Study, 40-y – 40-year programme, VHM&PP – Vorarlberg Health Monitoring and Prevention Programme, F/U – follow-up, PCa – prostate cancer, SD – standard deviation.

Table S3: Clinical characteristics of the 3,820 incident prostate cancer cases in the Swedish cohorts which are recorded in the Swedish National Prostate Cancer Register.

|  | **Total**  **(N=3,820)** |
| --- | --- |
| **Age at PCa diagnosis [years], mean (SD)** | 68.2 (7.1) |
| **PCa risk category, n (%)^1^** |  |
| **Localised low-risk** | 1,145 (30.0%) |
| **Localised intermediate-risk** | 1,176 (30.8%) |
| **Localised high-risk** | 755 (19.8%) |
| **Regionally metastatic/locally advanced** | 214 (5.6%) |
| **Distant metastases** | 437 (11.4%) |
| **Missing** | 93 (2.4%) |
| **Charlson comorbidity index, n (%)^2^** |  |
| **0 (no comorbidity)** | 3,074 (80.5%) |
| **1 (mild comorbidity)** | 364 (9.5%) |
| **≥2 (severe comorbidity)** | 256 (6.7%) |
| **Missing** | 126 (3.3%) |
| **Local clinical tumour stage, n (%)** |  |
| **T0** | 32 (0.8%) |
| **T1a, T1b** | 183 (4.8%) |
| **T1c** | 1,752 (45.9%) |
| **T1 unspecified** | 2 (0.1%) |
| **T2** | 1,183 (31.0%) |
| **T3, T4** | 620 (16.3%) |
| **TX^3^** | 47 (1.2%) |
| **Missing^3^** | 1 (<0.1%) |
| **Lymph node metastasis, n (%)** |  |
| **N0, no lymph node metastasis** | 673 (17.6%) |
| **N1, lymph node metastasis** | 130 (3.4%) |
| **Nx, no lymph node extirpation performed^3^** | 3,005 (78.7%) |
| **Missing^3^** | 12 (0.3%) |
| **Bone metastasis, n (%)** |  |
| **M0, no bone metastasis** | 2,222 (58.2%) |
| **M1, bone metastasis** | 331 (8.7%) |
| **Mx, no bone scan performed^3^** | 1,257 (32.9%) |
| **Missing^3^** | 10 (0.3%) |
| **Tumour differentiation, n (%)^4^** |  |
| **Low grade** | 233 (6.1%) |
| **Intermediate grade** | 375 (9.8%) |
| **High grade** | 175 (4.6%) |
| **Gx^3^** | 29 (0.8%) |
| **Missing^3^** | 3008 (78.7%) |
| **PSA at diagnosis [ng/mL], n (%)** |  |
| **<4** | 280 (7.3%) |
| **4-9.9** | 1,640 (42.9%) |
| **10-49.9** | 1,345 (35.2%) |
| **≥50** | 474 (12.4%) |
| **Missing** | 81 (2.1%) |
| **Primary treatment, n (%)^5^** |  |
| **Conservative** | 1,005 (26.3%) |
| **Curative** | 1,916 (50.2%) |
| **Non-curative** | 844 (22.1%) |
| **Dead before treatment decision** | 13 (0.3%) |
| **Missing** | 42 (1.1%) |
| **PCa deaths, n (%)** | 453 (11.9%) |

^1^Localised low-risk: T1-2, Gleason score 2-6 and PSA <10 ng/mL; localised intermediate-risk: T1-2, Gleason score 7 and/or PSA 10 to <20 ng/mL; localised high-risk: T3 and/or Gleason score 8-10 and/or PSA 20 to <50 ng/mL; regionally metastatic/locally advanced: T4 and/or N1 and/or PSA 50 to <100 ng/mL in the absence of distant metastases; distant metastases: M1 and/or PSA ≥100 ng/mL.

^2^Based on discharge diagnoses in the Swedish Patient Register.

^3^Tx, Nx, Mx and Gx imply that these were never measured, and the reason for missing data is unknown.

^4^Classified according to Gleason grading or WHO grade into low grade (Gleason score 2-6 or WHO grade 1), intermediate grade (Gleason score 7 or WHO grade 2) or high grade (Gleason score ≥8 or WHO grade 3).

^5^Conservative treatment includes watchful waiting and active surveillance; curative treatment includes radical prostatectomy and radiotherapy; non-curative treatment includes all androgen deprivation therapies (orchiectomy, GnRH agonists and antagonists) and antiandrogens.

Abbreviations: PCa – prostate cancer, PSA – prostate-specific antigen, SD – standard deviation.

Table S4. Mediation analysis of the effect of BMI on PCa death without correction for regression dilution ratio.

|  | **Model^1^** | **PCa deaths/N** | **Total effect HR (95% CI)^2^** | **Natural direct effect HR (95% CI)^2^** | **Natural indirect effect HR (95% CI)^2^** | **Proportion mediated % (95% CI)** |
| --- | --- | --- | --- | --- | --- | --- |
| **PCa-case only analysis (time from date of diagnosis)** | | | | | | |
| **All cases** | Model 1 | 1,784/11,760 | 1.19 (1.10 to 1.28) | 1.15 (1.06 to 1.25) | 1.03 (1.00 to 1.06) | 18% (0% to 35%) |
| **Cases with fasting samples** | Model 1 | 958/7,794 | 1.20 (1.08 to 1.32) | 1.12 (1.00 to 1.24) | 1.07 (1.03 to 1.11) | 39% (9% to 68%) |
| **Cases diagnosed with PCa within 10 years after baseline^3^** | Model 1 | 695/5,688 | 1.20 (1.08 to 1.34) | 1.16 (1.03 to 1.30) | 1.04 (1.00 to 1.08) | 21% (-5% to 48%) |
| **Swedish cases in the NPCR** | Model 1 | 453/3,820 | 1.10 (0.95 to 1.28) | 1.03 (0.88 to 1.21) | 1.06 (1.01 to 1.13) | 65% (-51% to 180%) |
| **Swedish cases in the NPCR** | Model 2 | 453/3,820 | 1.10 (0.94 to 1.29) | 1.04 (0.88 to 1.22) | 1.06 (1.00 to 1.12) | 59% (-49% to 167%) |
| **Full cohort analysis (age at PCa-specific death)** | | | | | | |
| **All men** | Model 3 | 1,784/259,884 | 1.15 (1.07 to 1.24) | 1.14 (1.05 to 1.23) | 1.01 (0.98 to 1.04) | 6% (-13% to 25%) |
| **Men with fasting samples** | Model 3 | 958/148,541 | 1.14 (1.04 to 1.26) | 1.11 (1.00 to 1.22) | 1.03 (1.00 to 1.07) | 25% (-6% to 56%) |
| **Swedish cohorts with cases in the NPCR** | Model 3 | 453/68,337 | 1.08 (0.94 to 1.24) | 1.03 (0.89 to 1.20) | 1.04 (0.99 to 1.10) | 55% (-69% to 179%) |

^1^Model 1: adjusted for age at PCa diagnosis, body mass index, smoking status, fasting status, and stratified on cohort and birth decade. Model 2: adjusted for the same variables as in Model 1, plus additionally adjusted for country of birth, education at the time of diagnosis, income closest to diagnosis, source of income closest to diagnosis, civil status closest to diagnosis, Charlson comorbidity index, primary treatment for prostate cancer, and prostate cancer risk category. Model 3: adjusted for age at study entry, smoking status, fasting status, and stratified on cohort and birth decade.

^2^HRs (reported per 5-kg/m^2^ increase in BMI) were estimated according to the two-stage regression method proposed by VanderWeele, with time since PCa diagnosis as the underlying time scale for the PCa-case only analysis, and attained age as the underlying time scale for the full cohort analysis. 95% confidence intervals (CIs) were computed using the Delta method. No correction for regression dilution ratio was performed. Effects corrected for regression dilution ratio are shown in Table 3 and Table 4.

^3^In case that the baseline visit occurred more than 10 years prior to PCa diagnosis, the first follow-up examination falling within the 10 year time window before PCa diagnosis, if available, was used.

Abbreviations: BMI – body mass index, CI – confidence interval, HR – hazard ratio, NPCR - National Prostate Cancer Register, PCa – prostate cancer.

**
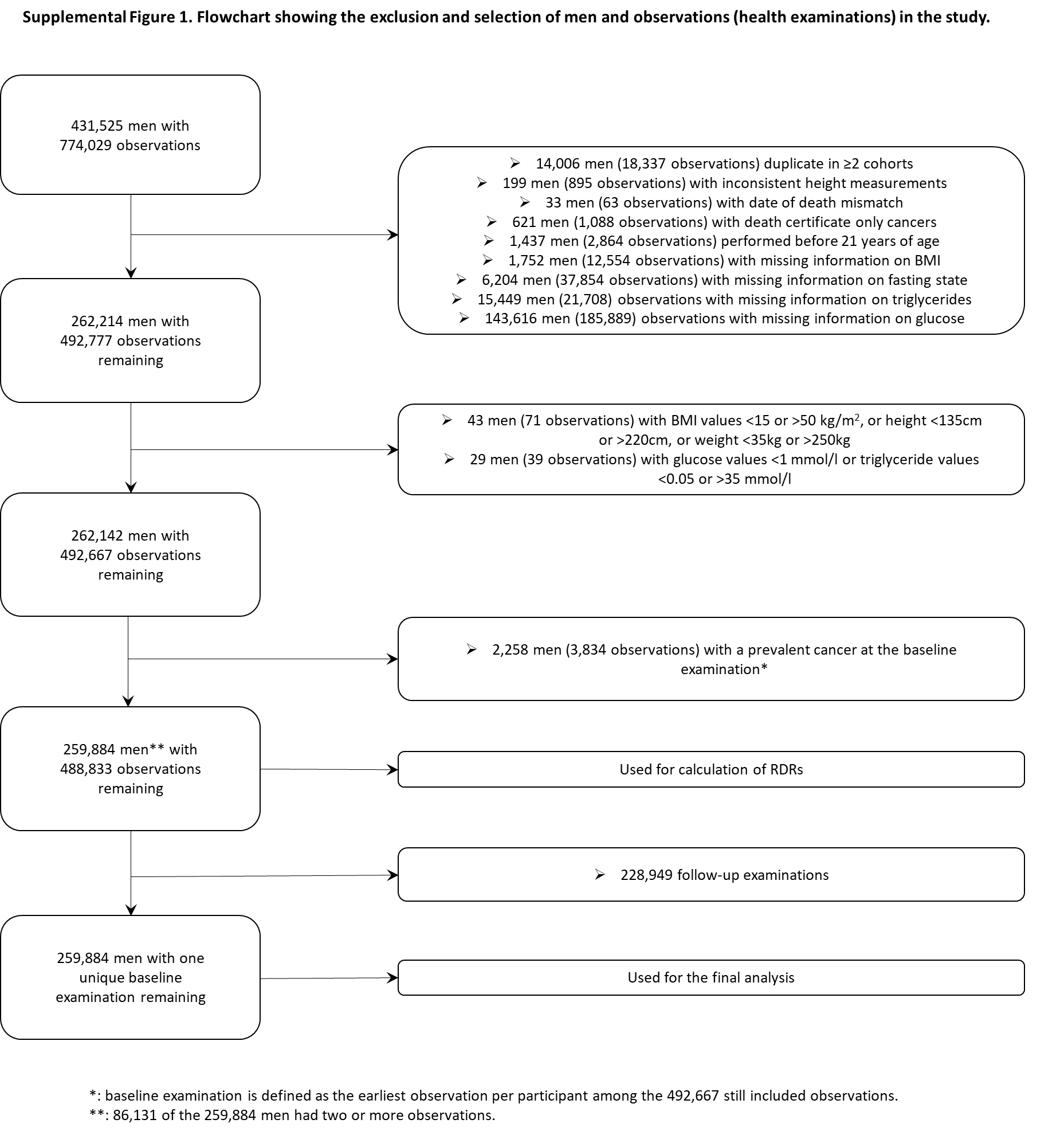
**

Figure S1. Flowchart showing the exclusion and selection of men and observations (health examinations) in the study.

**
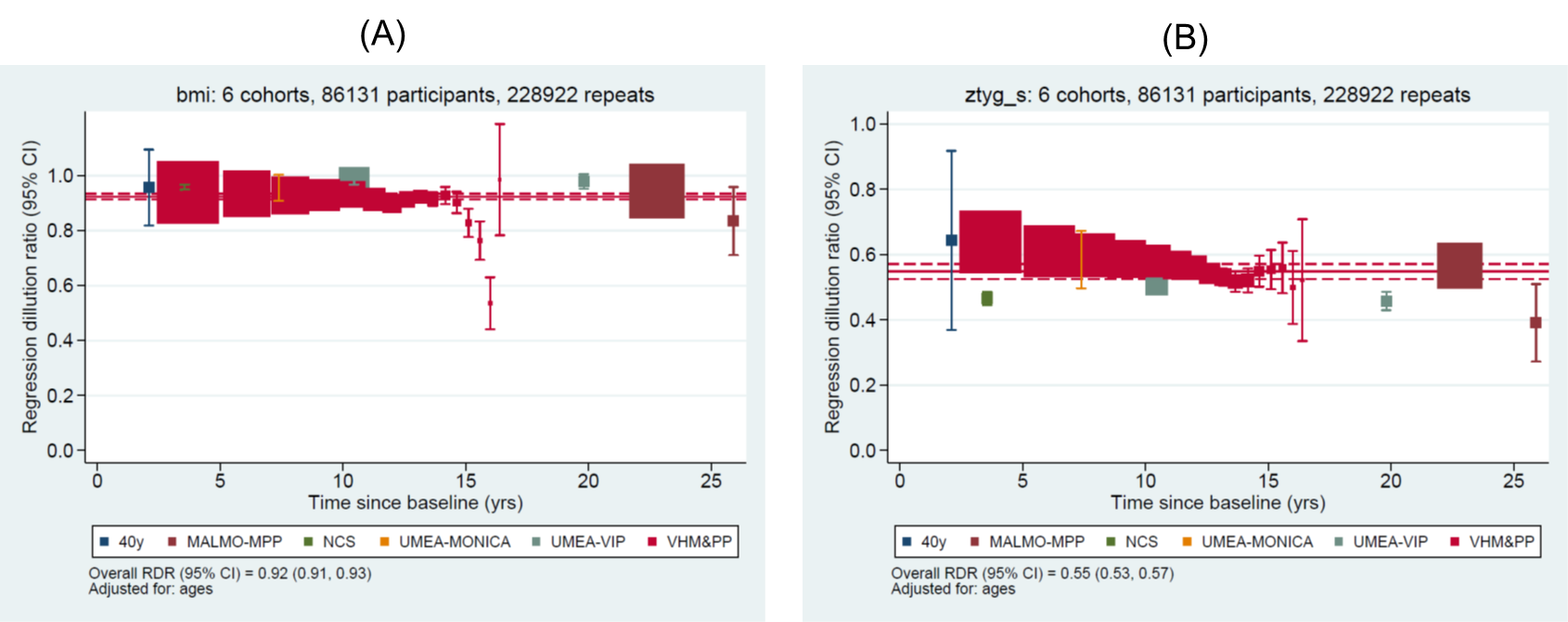
**

Figure S2. Overall and cohort and time-specific regression dilution ratios (95% confidence intervals) of (A) body mass index and (B) the TyG index^1^.

^1^z-transformed TyG index values, transformation performed separately for cohort and fasting status (<8h vs. ≥8h).
